# Advanced CT measures of coronary artery disease with intermediate stenosis in patients with severe aortic valve stenosis

**DOI:** 10.1007/s00330-023-10549-8

**Published:** 2024-01-08

**Authors:** Marcel C. Langenbach, Isabel L. Langenbach, Borek Foldyna, Victor Mauri, Konstantin Klein, Sascha Macherey-Meyer, Sebastian Heyne, Max Meertens, Samuel Lee, Stephan Baldus, David Maintz, Marcel Halbach, Matti Adam, Hendrik Wienemann

**Affiliations:** 1 Faculty of Medicine and University Hospital Cologne, Institute for Diagnostic and Interventional Radiology, University of Cologne, Kerpener Strasse 62, Cologne, 50937 Germany; 2 Cardiovascular Imaging Research Center, Department of Radiology, Massachusetts General Hospital, Harvard Medical School, 165 Cambridge Street, Suite 400, Boston, MA 02114 USA; 3 Faculty of Medicine and University Hospital Cologne, Clinic III for Internal Medicine, University of Cologne, Kerpener Strasse 62, 50937 Cologne, Germany

**Keywords:** Coronary artery disease, Tomography (X-ray computed), Fractional flow reserve (myocardial), Coronary angiography, Aortic valve stenosis

## Abstract

**Background:**

Coronary artery disease (CAD) and severe aortic valve stenosis (AS) frequently coexist. While pre-transcatheter aortic valve replacement (TAVR) computed tomography angiography (CTA) allows to rule out obstructive CAD, interpreting hemodynamic significance of intermediate stenoses is challenging. This study investigates the incremental value of CT-derived fractional flow reserve (CT-FFR), quantitative coronary plaque characteristics (e.g., stenosis degree, plaque volume, and composition), and peri-coronary adipose tissue (PCAT) density to detect hemodynamically significant lesions among those with AS and CAD.

**Materials and methods:**

We included patients with severe AS and intermediate coronary lesions (20–80% diameter stenosis) who underwent pre-TAVR CTA and invasive coronary angiogram (ICA) with resting full-cycle ratio (RFR) assessment between 08/16 and 04/22. CTA image analysis included assessment of CT-FFR, quantitative coronary plaque analysis, and PCAT density. Coronary lesions with RFR ≤ 0.89 indicated hemodynamic significance as reference standard.

**Results:**

Overall, 87 patients (age 77.9 ± 7.4 years, 38% female) with 95 intermediate coronary artery lesions were included. CT-FFR showed good discriminatory capacity (area under receiver operator curve (AUC) = 0.89, 95% confidence interval (CI) 0.81–0.96, *p* < 0.001) to identify hemodynamically significant lesions, superior to anatomical assessment, plaque morphology, and PCAT density. Plaque composition and PCAT density did not differ between lesions with and without hemodynamic significance. Univariable and multivariable analyses revealed CT-FFR as the only predictor for functionally significant lesions (odds ratio 1.28 (95% CI 1.17–1.43), *p* < 0.001). Overall, CT-FFR ≤ 0.80 showed diagnostic accuracy, sensitivity, and specificity of 88.4% (95%CI 80.2–94.1), 78.5% (95%CI 63.2–89.7), and 96.2% (95%CI 87.0–99.5), respectively.

**Conclusion:**

CT-FFR was superior to CT anatomical, plaque morphology, and PCAT assessment to detect functionally significant stenoses in patients with severe AS.

**Clinical relevance statement:**

CT-derived fractional flow reserve in patients with severe aortic valve stenosis may be a useful tool for non-invasive hemodynamic assessment of intermediate coronary lesions, while CT anatomical, plaque morphology, and peri-coronary adipose tissue assessment have no incremental or additional benefit. These findings might help to reduce pre-transcatheter aortic valve replacement invasive coronary angiogram.

**Key Points:**

*• Interpreting the hemodynamic significance of intermediate coronary stenoses is challenging in pre-transcatheter aortic valve replacement CT.*

*• CT-derived fractional flow reserve (CT-FFR) has a good discriminatory capacity in the identification of hemodynamically significant coronary lesions.*

*• CT-derived anatomical, plaque morphology, and peri-coronary adipose tissue assessment did not improve the diagnostic capability of CT-FFR in the hemodynamic assessment of intermediate coronary stenoses.*

## Introduction

Degenerative aortic valve stenosis (AS) frequently coexists with concomitant coronary artery disease (CAD) [1, 2]. The current European and North American guidelines recommend coronary CT angiography (CTA) or invasive coronary angiography (ICA) before transcatheter aortic valve replacement (TAVR) in patients with AS [3, 4]. Physiology-guided coronary revascularization is recommended for anatomical intermediate coronary stenosis [5]. For the invasive hemodynamic coronary assessment, resting full-cycle ratio (RFR) is one promising non-hyperemic resting pressure-derived index measured over the whole cardiac cycle [6].

CTA has a high negative predictive value for excluding obstructive CAD [7, 8]. However, severe coronary plaque burden and calcifications limit the evaluation patients with AS [9]. CT-derived fractional flow reserve (CT-FFR), based on computational fluid dynamics modeling, is a noninvasive method that provides additional information on lesions’ hemodynamic significance [10]. CT-FFR outperforms CTA stenosis assessment for detecting flow-limiting lesions in AS patients with invasive anatomical or physiological assessment as a reference standard [11–14].

CT analysis, including measures of coronary plaque volume and composition, and peri-coronary adipose tissue (PCAT) density, has shown incremental prognostic value to qualitative coronary assessment [15]. For instance, necrotic core volume, an index of plaque vulnerability, might be associated with the hemodynamic relevance of intermediate coronary lesions [16]. Additionally, PCAT density, an imaging marker of vascular inflammation, is different between three stages of CAD (no disease, stable CAD, and acute myocardial infarction) [17, 18].

However, it remains unknown whether these advanced CT measures help to detect hemodynamic significance of intermediate coronary lesions in patients with severe AS undergoing TAVR.

Hence, the study aims to elucidate the diagnostic performance of coronary CTA-derived, CT-FFR, CT-based plaque morphology, and PCAT density using RFR as the reference standard in patients with severe AS and intermediate coronary stenosis.

## Methods

### Patient population

This retrospective study included patients with a confirmed diagnosis of severe AS referred for pre-TAVR assessment, including both CTA and ICA, during the period from August 2016 to April 2022. To be eligible for study inclusion, patients required at least one intermediate coronary artery stenosis (diameter stenosis ranging from 20 to 80%), determined by CTA and ICA. Furthermore, patients were required to have undergone physiological lesion assessment using RFR of at least the intermediate stenotic lesion. All affected vessels were considered in this study.

Exclusion criteria encompassed patients undergoing an ICA at external medical facilities, a time interval exceeding 3 months between CT and ICA, history of prior coronary artery bypass graft surgery, recent myocardial infarction within the preceding 3 months, chronic total occlusions, employment of a different CT system for pre-TAVR assessment, CT scan after PCI, non-standardized pre-TAVR CT scan protocol, and instances with diagnostically inadequate image quality. These exclusion criteria were meticulously selected to facilitate an optimal alignment between CT findings and invasive assessments, thereby enhancing the validity of comparisons.

In the case of multivessel disease, demographic and clinical data were evaluated per patient. The data supporting this study’s findings are available upon reasonable request. The institutional review board approved the study (IRB, 22–1154) with a waiver of written informed consent due to the retrospective design. The study was carried out in conformity with the Declaration of Helsinki.

### Acquisition and analysis of CT datasets (CT-TAVR image reconstruction)

Data were acquired using a third-generation dual-source 2 × 192-slice CT system (Somatom Force, Siemens Healthineers). The protocol followed the recommendations of the Society of Cardiovascular Computed Tomography (SCCT), adapted for local requirements [19, 20]. No premedication with beta-blockers or nitrates was applied before the CT acquisition. A retrospectively electrocardiogram-gated scan in cranial-caudal orientation examining the heart, the entire aorta, the iliac, and the common femoral arteries in the arterial phase was performed. For the CT imaging protocol, a tube current of 100 kV with dose modulation set for a quality reference of 300 mAs was used. Rotation time was 0.25 s; reconstructions were performed using a smaller heart-orientated field-of-view (FOV) with a slice thickness of 0.75 mm and an entire FOV with a 1-mm slice thickness for the complete scan. Coronary and cardiac analyses were performed using the cardiac-centered FOV. Patients received 60 mL iodinated contrast medium (ICM) (Imeron 400, Bracco Imaging S.p.A.) using a power injector (Accutron CT, Metron) with a standardized injection protocol. This contained a bolus of 60 mL ICM at an injection rate of 5 mL/s followed by a 40-mL saline chaser injected with 5 mL/s. The threshold for automatic initiation of the standardized TAVR CT protocol was 120 Hounsfield units (HU) in the ascending aorta.

Two radiologists performed a semi-automated assessment of the coronary vessels with 4 and 6 years of experience (K.K., M.L.) in cardiac imaging in consensus. The readers were blinded to the invasive RFR results. For identification of the lesion, readers were furnished with precise lesion locations, as per the 18-segment coronary model delineated in the SCCT guidelines [21], and using the available ICA images.

### CT-FFR analysis

The CT dataset was processed using a validated stand-alone CT-FFR software prototype (cFFR version 3.0, Siemens Healthineers; currently not commercially available) [22]. This software approach has been well described in previous recommendations, also for patients with AS [13, 23, 24]. In brief, CT-FFR requires a 3D anatomical model of the coronary arteries, the formulation of a mathematical representation of coronary physiology to establish boundary conditions encompassing parameters like cardiac output, aortic pressure, and microcirculatory resistance, and the subsequent utilization of numerical techniques to solve the governing fluid dynamics equations derived from fundamental physics principles. This amalgamation of anatomical precision, physiological insights, and fluid dynamics expertise collectively empowers the computation of both coronary flow and pressure dynamics [23]. The readers performed a semi-automated manual adapted assessment and definition of the coronary artery tree, centerlines, vessel lumen, and stenosis. CT-FFR values were measured 10 mm distal of the lesion of interest, according to previous recommendations [25]. Applicability was limited by a minimal luminal diameter of 1.5 mm. The left ventricle myocardial mass was automatically determined in the CT images to estimate the resting total coronary blood flow. Ischemic obstructive CAD was defined with a lesion-specific CT-FFR value ≤ 0.80.

### Quantitative coronary plaque CT measurements

Semi-automated quantitative evaluation of plaque parameters, including metric measurements and composition, was performed using a qualified and validated investigational on-site software (Medis Suite CT Plaque Analysis Medical Imaging Systems, V3.2) and the CT datasets [26, 27]. The readers identified the invasively assessed intermediate coronary lesion for quantitative CT evaluation utilizing the same approach as for the CT-FFR evaluation. The target lesion was the region with atherosclerotic alterations between non-affected proximal and distal parts. All relevant coronary lesions were segmented to assess the plaque compositions in detail. Metric measurements comprised the maximum diameter stenosis, maximum area stenosis, and lesion length. The Coronary Artery Diseases-Reporting & Data System (CAD-RAD) was used to determine the degree of stenosis as follows: (1) 1–24%, (2) 25–49%, (3) 50–69%, (4) 70–99%, and (5) 100% [28]. We measured total, calcified, and noncalcified plaque volumes on a per-lesion level. Atherosclerotic plaque volume was subclassified by radiodensity, using predefined validated intensity cutoffs for dense calcium (≥ 351 HU), fibrous (131–350 HU), fibro-fatty (31–130 HU), and necrotic-core (− 30 to 30 HU) [29].

### Assessment of peri-coronary adipose tissue density

We measured the PCAT density for the affected vessel according to the previously described standard approach in PCAT assessment using a validated software for research purposes (Medis Suite CT Plaque Analysis Medical Imaging Systems, V3.2) [17, 30]. A region of interest was circled radially outwards from the outer vessel wall within a radial distance from the wall equal to the vessel diameter. The first 10 mm of the right coronary artery and the left main artery from the coronary ostium was spared to avoid the effects of the aortic wall [17, 31]. Local vascular inflammation will increase PCAT density with less negative HU values (closer to − 30 HU). PCAT density was calculated using a voxel-by-voxel analysis within the segmented perivascular cylindrical space.

### Pressure wire assessment

Coronary angiography was conducted according to conventional clinical practice using the PressureWireTM X Guidewire (Abbott Vascular Inc.) for intracoronary pressure measurements according to the manufacturer’s instructions. RFR was measured directly (Quantien System v.1.12; Abbott Vascular Inc.) or calculated retrospectively in a core lab based on invasive FFR data (CoroLab; Coroventis Research AB). RFR is considered diagnostically equivalent to instantaneous wave-free ratio (IFR) in diagnostic accuracy, yet it holds an advantage in its impartial capacity to identify the lowest pressure ratio (*P*_distal_/*P*_aortic_) across the entire cardiac cycle [6]. This unbiased approach has the potential to unveil coronary stenoses of physiological significance that might remain undetected when evaluations are confined to specific segments of the cardiac cycle. RFR has been validated in the VALIDATE RFR study [6]. Hemodynamically significance was defined as RFR ≤ 0.89.

### Statistical analysis

Categorical variables are reported as frequencies and percentages and compared using chi-square statistics or Fisher’s exact test. Continuous variables are presented as median with interquartile range (IQR) or mean ± standard deviation. An unpaired Student *t*-test for normal distribution and Wilcoxon rank-sum test for asymmetric distribution were used to analyze differences in continuous variables. The association between CT-FFR, quantitative coronary plaque measurements, and invasive FFR was quantified using Spearman’s correlation coefficients. Correlation coefficients of < 0.2 were classified as very weak, 0.2 to < 0.40 as weak, 0.40 to < 0.60 as moderate, 0.6 to < 0.80 as strong, and 0.8 to 1 as very strong. The agreement was assessed with Bland–Altman analysis.

Receiver operating characteristic (ROC) analysis was used to calculate the area under the curve. The sensitivity, specificity, and calculation of positive and negative predictive values of CT-FFR for invasive RFR prediction were calculated. Univariable and multivariable logistic regression analyses were used to calculate odds ratios (OR) and 95% confidence intervals (CI) of functional lesions ischemia defined as invasively measured RFR ≤ 0.89 as a binary endpoint. In the multivariable model, we adjusted for maximum diameter stenosis, maximum lumen area stenosis, lesion length, PCAT density, and necrotic core volume. A *p* value of < 0.05 was considered statistically significant. All statistical analyses were performed with R 4.2.1 (R Foundation for Statistical Computing) or Stata 17.1 (College Station).

## Results

### Baseline characteristics

**Fig. 1:**
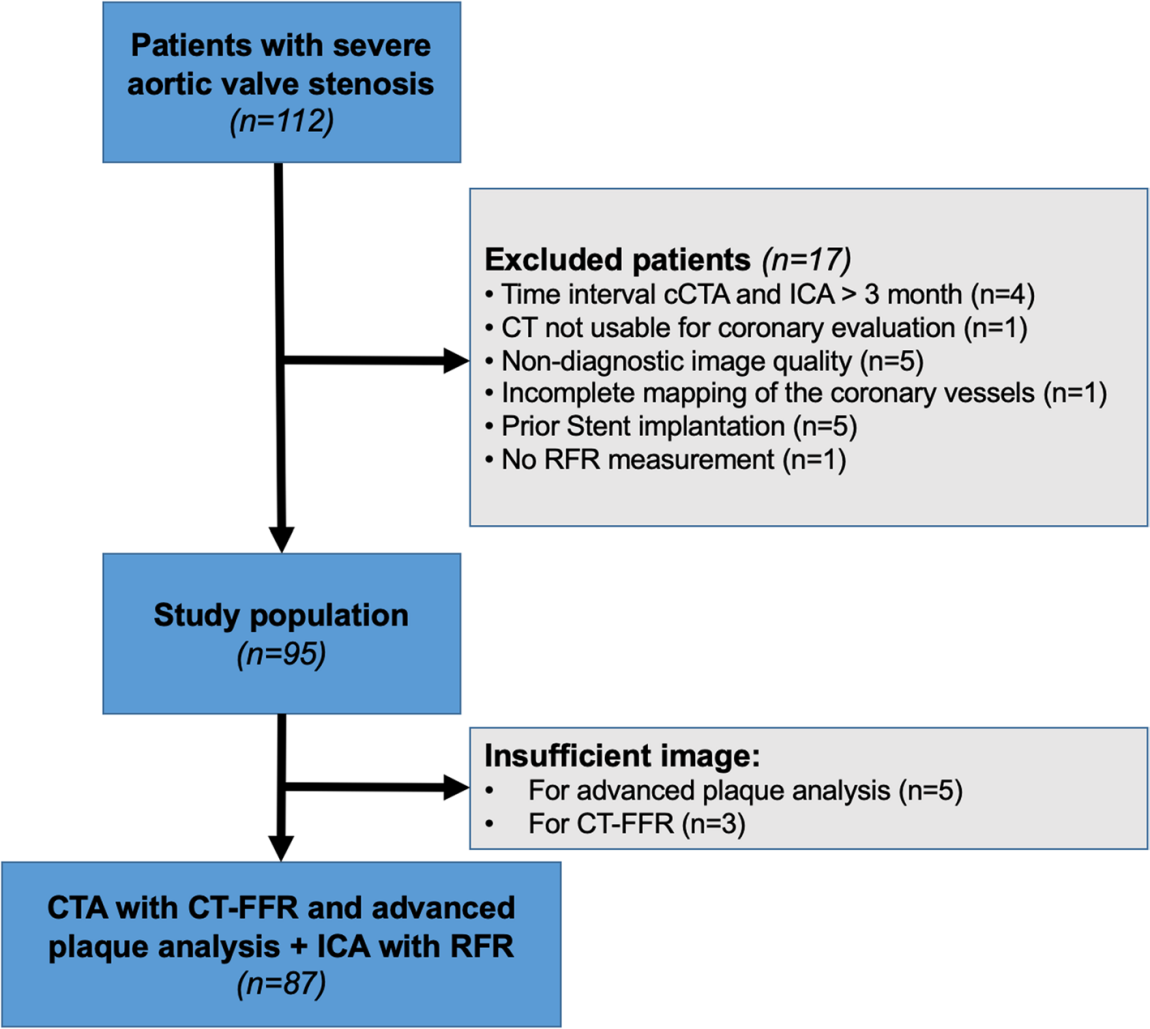
Patient flowchart. CTA, CT-angiography; CT-FFR, fractional flow reserve derived from coronary computed tomography angiography; RFR, Resting full-cycle ratio

In total, 87 patients with 95 intermediate lesions were included (Fig. 1). The mean patient age was 77.9 ± 7.4 years, 38% were females, and the mean aortic valve gradient was 45.9 ± 13.8 mmHg. Overall, the cohort presented a high prevalence of diabetes mellitus, hypertension, impaired kidney function, atrial fibrillation, and almost normal weight according to body mass index, as displayed in Table 1. The heart rate during the CT examination was 73 beats per minute (IQR 63–83), with no difference between the two groups (*p* = 0.62). Also, the radiation dose with a mean of 289.0 mGy*cm (IQR 213.5–461.5) showed no significant difference (*p* = 0.14).

**Table 1:** Patient baseline characteristics

	Overall, *N* = 87	RFR > 0.89, *N* = 48	RFR ≤ 0.89, *N* = 39	*p* value
Demographics				
Female	33 (38%)	18 (38%)	15 (38%)	0.93
Age, years	77.86 ± 7.43	77.75 ± 7.28	78.00 ± 7.71	0.88
Body mass index, kg/m^2^	26.10 (24.10–29.68)	26.27 (24.17–30.13)	25.66 (23.79–29.16)	0.34
Cardiovascular risk factors				
Diabetes mellitus	23 (26%)	15 (31%)	8 (21%)	0.26
Hypertension	66 (76%)	37 (77%)	29 (74%)	0.77
Hyperlipidemia	33 (38%)	17 (35%)	16 (41%)	0.59
Peripheral artery disease	9 (10%)	7 (15%)	2 (5.1%)	0.18
Former or current smoker	21 (24%)	13 (27%)	8 (21%)	0.48
Family history of coronary artery disease	11 (13%)	7 (15%)	4 (10%)	0.75
Atrial fibrillation	26 (30%)	15 (31%)	11 (28%)	0.76
Chronic obstructive pulmonary disease	5 (6%)	3 (6%)	2 (5%)	> 0.99
Serum biomarkers				
GFR (mL/min/1.73 m^2^)	61.19 (49.64–79.98)	59.89 (46.32–81.04)	61.26 (52.79–79.07)	0.61
GFR < 60 (mL/min/1.73 m^2^)	42 (48%)	25 (52%)	17 (44%)	0.43
Hemoglobin, g/dL	12.87 (1.93)	12.99 (2.11)	12.72 (1.70)	0.27
Echocardiographic parameters				
Mean aortic valve gradient, mmHg	45.86 ± 13.73	45.02 ± 14.11	46.90 ± 13.36	0.53
Left ventricular ejection fraction				> 0.99
≥ 50%	70 (80%)	39 (81%)	31 (79%)	
31–49	13 (15%)	7 (15%)	6 (15%)	
≤ 30%	4 (4.6%)	2 (4.2%)	2 (5.1%)	
Aortic regurgitation				0.21
None–trace	72 (83%)	42 (88%)	30 (77%)	
Mild	7 (8%)	4 (8%)	3 (8%)	
Moderate–severe	8 (9%)	2 (4%)	6 (15%)	
CAD-RADS classification				0.31
1	6 (7%)	5 (10%)	1 (3%)	
2	32 (37%)	18 (38%)	14 (36%)	
3	42 (48%)	23 (48%)	19 (49%)	
4	7 (8%)	2 (4%)	5 (13%)	

All data are mean ± standard deviation, median (Q1–Q3), or absolute number (percentage)

*CAD-RADS*, Coronary Artery Disease Reporting And Data System; *CT*, computed tomography; *RFR*, resting full-cycle ratio; *eGFR*, estimated glomerular filtration rate

### Coronary stenosis severity and physiological assessment

Sixty-one (64.0%) of the interrogated lesions were in the left anterior descending arteries (LAD). CTA revealed an overall mean stenosis diameter of 50.7% (IQR 40.6–60.7%). Forty-three lesions (45.0%) were measured with a luminal diameter stenosis between 50 and 69%. Median CT-FFR was 0.85 (IQR 0.77–0.91), with 37.0% of CT-FFR measurements being ≤ 0.80. Table 2 displays the detailed vessel characteristics. Median invasive RFR was 0.90 (IQR 0.87–0.95), and functionally significant stenosis (RFR ≤ 0.89) was observed in 42 (44.2%) vessels.

**Table 2:** Lesion characteristics

	Overall, *N* = 95	RFR > 0.89,* N* = 53	RFR ≤ 0.89, *N* = 42	*p* value
RFR	0.90 (0.87–0.95)	0.94 (0.92–0.98)	0.86 (0.82–0.88)	** < 0.001**
CT-derived lesion characteristics				
Proximal lesion	47 (49%)	27 (51%)	20 (48%)	0.75
Location				**0.030**
Left anterior descending	61 (64%)	27 (51%)	34 (81%)	
Left circumflex	24 (25%)	18 (34%)	6 (14%)	
Right coronary artery	8 (8%)	6 (11%)	2 (5%)	
Left main	2 (2%)	2 (4%)	0 (0%)	
Max. diameter stenosis, %	50.68 (40.57–60.69)	49.00 (37.35–58.91)	51.73 (43.46–64.44)	0.19
Max. diameter stenosis, cat				0.27
1–24%	9 (10%)	7 (13%)	2 (5%)	
25–49%	36 (38%)	21 (40%)	15 (36%)	
50–69%	43 (45%)	23 (43%)	20 (48%)	
70–99%	7 (7%)	2 (4%)	5 (12%)	
Max. area stenosis, %	76 (64–85)	74 (61–83)	77 (66–87)	0.20
Lesion length, mm	10.85 (7.69–17.44)	10.50 (7.50–13.31)	13.30 (8.30–19.54)	0.069
Volume and composition				
Total volume, mm^3^	87.73 (45.75–164.33)	85.39 (47.11–138.20)	111.04 (45.16–191.81)	0.37
Dense calcium volume, mm^3^	51.44 (14.72–101.24)	49.00 (13.17–65.36)	59.52 (16.85–135.06)	0.19
Noncalcified plaque volume	33.95 (21.12– 61.86)	33.64 (21.52– 60.63)	37.84 (20.23– 64.99)	0.89
Fibrotic plaque, mm^3^	23.75 (14.23–46.45)	21.44 (14.27–44.85)	29.64 (13.44–51.15)	0.37
Fibrofatty plaque, mm^3^	4.94 (2.47–9.12)	5.15 (2.58–9.16)	4.42 (2.44–8.98)	0.65
Necrotic core volume, mm^3^	3.17 (1.05–8.26)	3.56 (0.88–8.14)	2.44 (1.08–8.58)	0.85
Functional analysis				
CT-FFR	0.85 (0.77–0.91)	0.89 (0.85–0.94)	0.76 (0.72–0.79)	** < 0.001**
CT-FFR ≤ 0.80	35 (37%)	2 (4%)	33 (79%)	** < 0.001**
Coronary inflammation				
PCAT density, HU	–83.91 (–90.37, –73.90)	–81.30 (–89.82, –70.76)	–84.96 (–90.95, –77.67)	0.31

All data are mean ± standard deviation, median (Q1–Q3), or absolute number (percentage)

*CT-FFR*, computed tomography derived fractional flow reserve; *PCAT*, peri-coronary adipose tissue; *RFR*, resting full-cycle ratio

**Fig. 2:**
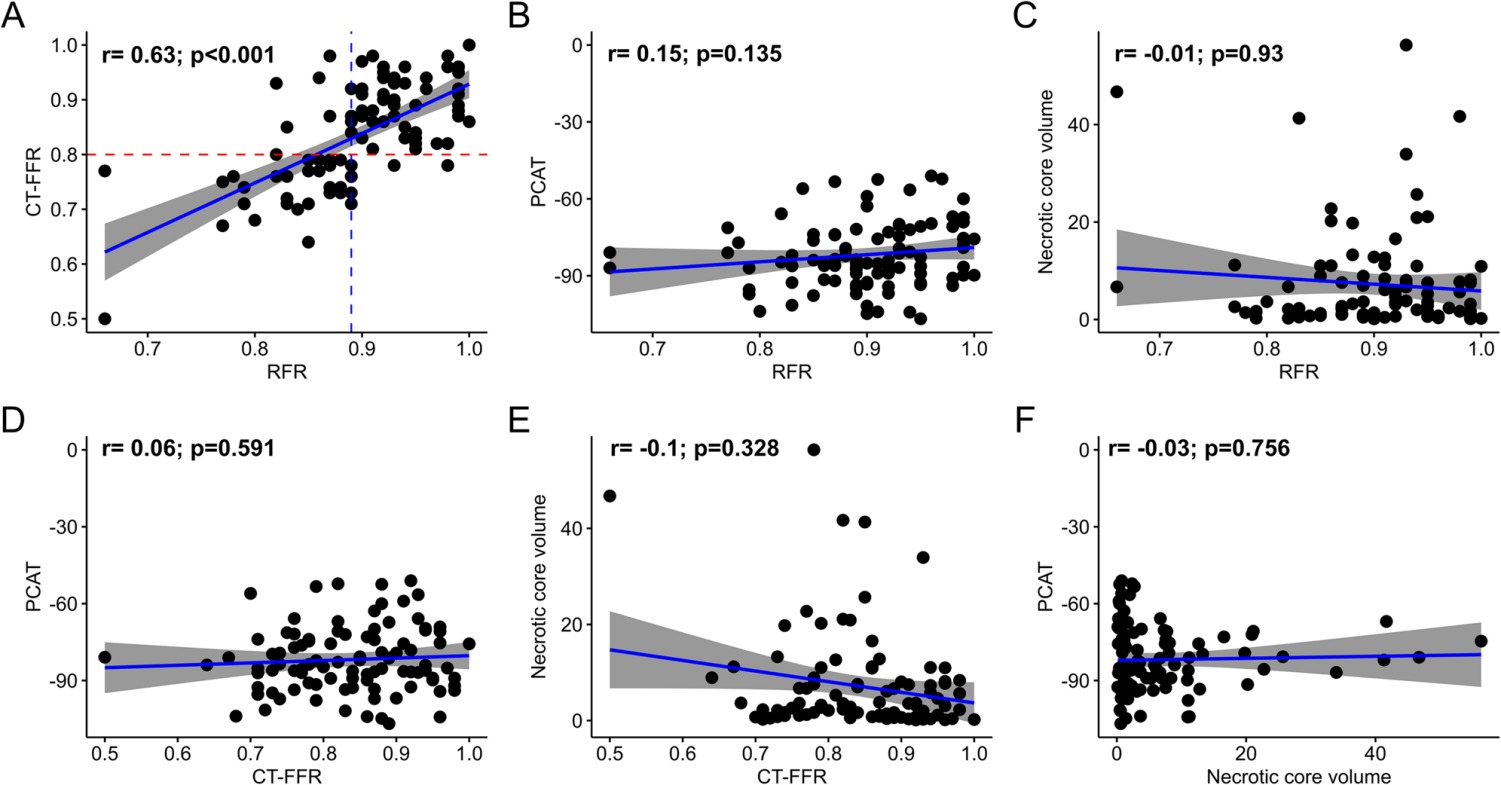
Correlation between CT-FFR, RFR, PCAT density, and necrotic core volume. Panels show a scatterplot for each variable with the coefficient correlation (*r*) derived from Spearman’s rho method, as appropriate. Correlation between RFR and CT-FFR (**A**), PCAT (**B**), and necrotic core volume (**C**). Correlation of CT-FFR with PCAT (**D**) and necrotic core volume (**E**). Correlation between necrotic core volume and PCAT (**F**). CT-FFR, fractional flow reserve derived from computed tomography angiography, diameter stenosis from computed tomography; PCAT, peri-coronary adipose tissue; RFR, resting full-cycle ratio

A good correlation (*r* = 0.63 (95%CI 0.495–0.746),* p* < 0.001) and agreement (mean difference 0.06, limits of agreement –0.075 to 0.198) between CT-FFR and RFR were found (Fig. 2a). Overall, CT-FFR showed a diagnostic accuracy, sensitivity, specificity, positive predictive value, and negative predictive value of 88.4% (95%CI 80.2–94.1%), 78.5% (95%CI 63.2–89.7%), 96.2% (95%CI 87.0–99.5%), 94.3% (95%CI 80.8–99.3%), and 85.0% (95%CI 73.4–92.9%) in predicting RFR ≤ 0.89.

### Intravascular plaque assessment and PCAT density

**Fig. 3:**
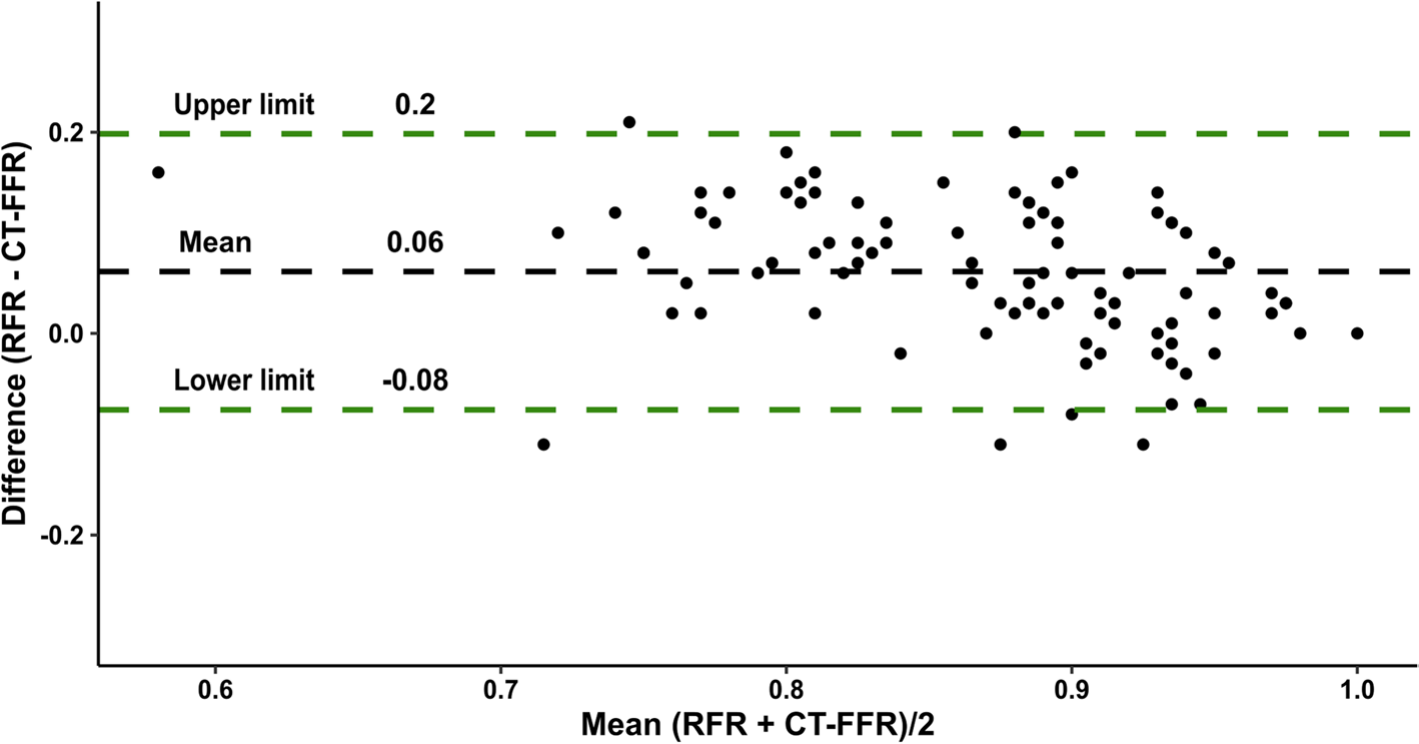
Bland–Altman plot. Agreement among CT-Fractional Flow Reserve and Resting full-cycle ratio. Mean difference between both techniques was 0.0614 (limits of agreement − 0.075 to 0.198). CT-FFR, fractional flow reserve derived from computed tomography, RFR, Resting full-cycle ratio

Hemodynamically relevant lesions (RFR ≤ 0.89) showed a non-significant trend for higher diameter stenosis, higher lumen area stenosis, and longer lesion lengths than lesions without ischemia. Moreover, the differences in metric plaque measurements and plaque composition, including necrotic core volume, between lesions with or without functional ischemia were non-significant (Table 2). PCAT density was also not statistically different in lesions without ischemia compared to ischemic lesions (− 81.3 (IQR − 89.82 to − 70.76) HU vs. − 84.96 (− 90.95 to − 77.67) HU, *p* = 0.31). Correlation analysis showed no significant correlation between RFR or CT-FFR and necrotic core volume or PCAT density (Fig. 2b–f). Agreement between RFR and CT-FFR using a Bland–Altman plot yielded a mean difference of 0.06 with 95% confidence limits between − 0.08 and 0.2 (Fig. 3).

### Diagnostic ability of lesion features, lesion-specific PCAT, and CT-FFR

According to the logistic regression analysis, only CT-FFR emerged as a predictor for RFR ≤ 0.89 with an odds ratio of 1.27 (95%CI, 1.17–1.41) in the univariable model. After adjustment for all co-variables in the multivariable model, CT-FFR remained a significant predictor (1.28 (95%CI, 1.17–1.43), *p* < 0.001) (Table 3).

**Table 3:** Logistic regression analyses for the prediction of resting full-cycle ratio ≤ 0.89

Characteristics	Univariable model		Multivariable model	
	OR (95%CI)	*p* value	OR (95%CI)	*p* value
CT-FFR	1.27 (1.17 to 1.41)	** < 0.001**	1.28 (1.17 to 1.43)	** < 0.001**
Max. diameter stenosis, %	0.98 (0.95 to 1.00)	0.10	0.96 (0.78 to 1.19)	0.73
Max. lumen area stenosis, %	0.98 (0.95 to 1.00)	0.10	1.04 (0.86 to 1.25)	0.69
Lesion length, mm	0.96 (0.91 to 1.00)	**0.041**	0.97 (0.90 to 1.03)	0.32
PCAT density, HU	1.02 (0.99 to 1.05)	0.27	1.02 (0.97 to 1.06)	0.47
Necrotic core volume, mm^3^	1.01 (0.97 to 1.05)	0.72	1.04 (0.99 to 1.12)	0.13

*CT-FFR*, computed tomography derived fractional flow reserve; *PCAT*, peri-coronary adipose tissue, *OR*, odds ratio

**Fig. 4:**
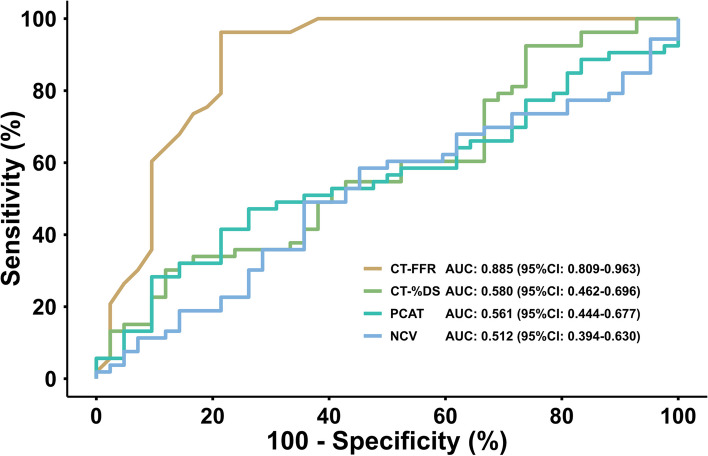
Receiver operating characteristic curves. Area under the curve in receiver operating characteristic analysis of CT-FFR, CT-%DS, PCAT density, and NCV to identify a resting full-cycle ratio ≤ 0.89. AUC, area under the curve; CT-FFR, fractional flow reserve derived from computed tomography angiography, diameter stenosis from computed tomography; CT-%DS, percent diameter stenosis from computed tomography; PCAT, peri-coronary adipose tissue; NCV, necrotic core volume

Additionally, the area under the ROC curve (AUC) demonstrated high diagnostic performance of CT-FFR regarding its ability to detect functionally significant stenosis, taking RFR as reference (AUC 0.885 (95% CI, 0.809–0.963)) (Fig. 4). Adding other CT-derived imaging features did not improve the area under the curve (CT-FFR + CTA: AUC 0.884 (95%CI, 0.807–0.960); CT-FFR + PCAT density AUC 0.888 (95%CI, 0.813–0.962); CT-FFR and necrotic core volume AUC 0.887 (95%CI, 0.809–0.965); CT-FFR and CTA and PCAT density and necrotic core volume AUC 0.881) 95%CI, 0.812–0.965)).

## Discussion

This study compared CT-FFR, CT-derived coronary plaque parameters, and PCAT density to diagnose ischemic CAD defined by RFR in patients with severe AS and intermediate coronary stenosis. To our knowledge, this was the first study investigating the applicability of coronary plaque features in assessing ischemic coronary lesions in patients with relevant AS and intermediate stenotic lesions. The main findings were that CT-FFR might improve the CTA’s diagnostic capability in diagnosing ischemic lesions in patients with severe AS. Intra- and extravascular CT parameters yielded no incremental benefit in identifying ischemic lesions. These results emphasize CT-FFR’s potential as a predictive tool for identifying cases requiring further invasive assessment or intervention. However, it is important to recognize that the clinical implications of our findings should be interpreted in conjunction with the broader clinical context, potential confounding factors, and limitations due to the limited sample size.

Initially, Michail et al reported a 76.7% diagnostic accuracy in a small-scale cohort for CT-FFR, taking FFR as the reference standard [24]. These results are supported by a previous proof-of-concept study from our group investigating CT-FFR in severe AS patients [11]. More recently, Gohmann et al and Peper et al showed in larger severe AS patient cohorts the feasibility of CT-FFR and its additive value to CTA for the correct classification of patients with morphological signs of obstructive CAD [13, 14]. However, in both studies, ICA was dominantly considered the reference standard, which was inferior to invasive FFR assessment to guide revascularization in the FAME study [32]. A body of evidence is growing for using non-hyperemic pressure ratios like the instantaneous wave-free ratio or RFR [6, 33, 34]. FFR and non-hyperemic pressure ratios are well correlated with CT-FFR [35, 36]. Aquino et al have shown that positive CT-FFR was independently associated with the occurrence of major adverse cardiac events in patients undergoing TAVR for severe AS [37].

Applying additional CT-derived imaging parameters might reduce the rate of false-negative findings. Various CT-derived features, including plaque composition and PCAT density, might improve the diagnostic accuracy of CTA and CT-FFR in detecting hemodynamically significant or vulnerable coronary lesions [15, 30, 38, 39].

Our results confirmed lesion classification based on metric parameters is of limited value [9]. The AUC for the diameter stenosis with 0.580 was below the expected result, though we only investigated intermediate stenoses. One reason might be extensive calcifications of the coronary arteries with blooming artifacts, leading to a reduced diagnostic capability of CAD by anatomic assessment alone in patients with AS [9, 40]. Further, we used a CT protocol without a dedicated acquisition of the coronary arteries. In conclusion, the additional evaluation of diameter stenosis to CT-FFR had no independent or incremental benefit.

Low-density or necrotic core volume showed no association with the diagnosis of RFR-defined ischemia. Sezer et al showed a significant correlation between necrotic core volume and invasive FFR [16]. In contrast, Naya et al found no significant association between plaque length, plaque composition by coronary CTA, and the extent of myocardial flow reserve [41]. We hypothesized an increased necrotic core volume as a factor with increased hemodynamic relevance. The altered plaque structure was assumed to influence the stenosis relevance, taking the study of Hell et al into consideration [42]. Based on our results, low-density plaque volume had no association with the prediction of RFR-defined ischemia. While the direct association between plaque characteristics and the hemodynamic significance of coronary lesions might not be evident within our cohort, it is crucial to acknowledge that this does not necessarily negate the potential link to adverse cardiac events. As demonstrated by Ferencik et al and Puchner et al, these studies have highlighted that certain plaque compositions and phenotypes are indeed linked to unfavorable outcomes [43, 44]. Although these findings have not been specifically replicated within a cohort of patients with AS, we can reasonably extrapolate their relevance to this population.

PCAT density has been identified as a surrogate for vascular and coronary inflammation. The association between PCAT density, plaque extent, and composition is ongoing research, as coronary inflammation leads to changes in the coronary vessel wall and the plaque composition [17, 31]. It has been demonstrated by Lin et al that PCAT attenuation reliably distinguishes different stages of CAD [18], and Wen et al found an improvement in the diagnostic capability of coronary CTA by adding PCAT density [45]. Further, Zhou et al showed that a PCAT radiomics model showed good prospects in predicting myocardial ischemia [46]. On the contrary, based on our study’s results, PCAT density does not allow as a surrogate for ischemic coronary lesions. A reason for this might be the use of contrast-enhanced images for PCAT assessment and the relatively small expected difference between hemodynamic significant vs. non-significant lesion (− 65.6 ± 5.9 vs. − 75.3 ± 5.4 HU). This is in line with previous studies using epicardial adipose tissue (EAT) instead of PCAT density. In a small sub-study, Mutathalay et al found no discriminator ability for EAT volume in ischemia and non-ischemia groups defined by computed tomographic perfusion imaging and invasive FFR [47]. Further, the multicenter study CORE 320 found no association between EAT volume and myocardial perfusion abnormalities [48]. In contrast to these results, a study by Brandt et al demonstrated that combining EAT volume with plaque quantification identified myocardial ischemia similar to CT-FFR [39]. PCAT density or EAT was measured with inherent potential errors. The expected difference between inflammatory changes and no inflammations is below 10 HU [17]. The density is often assessed in contrast-enhanced CT images. This issues a variable contrast accumulation and distribution in the fat tissue. Therefore, an assessment of unenhanced images might be favorable.

In addition to CT-FFR, the integration of dynamic CT perfusion in conjunction with coronary CT also holds promise for evaluating the hemodynamic significance of coronary lesions. This potential has been validated through the SPECIFIC trial, which achieved an 88% accuracy in detecting significant coronary stenosis, compared to the 78% accuracy achieved by CT for stenosis ≥ 50% [49]. The outcomes of this multicenter study align with Lu et al’s findings, where sensitivity and specificity reached 83% and 91%, surpassing the 82% sensitivity and 61% specificity obtained through coronary CTA alone [50]. Nonetheless, it is imperative to acknowledge that the utility of dynamic CT perfusion imaging is accompanied by several constraints, including heightened radiation exposure, extended scan duration, and the inherent challenge of precisely quantifying absolute myocardial blood flow [49].

Considering all aspects, CT-FFR could be used as a clinical tool to rule out hemodynamically relevant CAD in patients with intermediate coronary stenosis and sufficient image quality. Additional studies are needed to elucidate the clinical benefit of the CT-FFR fully and to secure a high accuracy. In addition, it should be mentioned that TAVR becomes a less risky intervention, shifting the requirement of revascularization toward a more selective approach in this increasingly heterogeneous population.

### Study limitations

This is a retrospective observational study with inherent limitations. First, the patient cohort was relatively small and investigated at a single academic hospital with a higher exclusion rate compared to large-scale studies [35, 51]. Second, we did not acquire a dedicated coronary CTA scan. A cardiac-centered FOV was used for the evaluation of the coronary arteries. The used CT-FFR software and the software kit for assessing coronary plaque composition and PCAT density are currently only available for research purposes. Third, the PCAT density assessment was performed utilizing contrast-enhanced images, consistent with prior investigations. However, the anticipated distinctions between inflamed and non-inflamed PCAT remain subtle. The application of contrast enhancement may impact the discernment of these disparities and obscure any alterations. Fourth, the high AUC for CT-FFR and positive odds ratio indicates a good predictive value for RFR-defined ischemia. However, the confidence intervals around these measurements remain broad, warranting caution when interpreting these measures interchangeably, and these measures are derived from a relatively modest sample size. Additionally, the analyzing radiologists could access the recorded ICA images, which could result in a potential bias for the CT-FFR measurements. Fifth, the reference standard used in the present study was the invasive RFR, while currently, most studies use FFR or instantaneous wave-free periods, but randomized data regarding these indices are lacking in patients with AS.

## Conclusion

Noninvasive CT-FFR assessed by routine pre-TAVR CT performed superior to quantitative metric measurements, plaque composition, and PCAT density assessment of coronary arteries in determining the hemodynamic relevance of intermediate stenotic coronary lesions defined by invasive RFR in patients with relevant AS. Quantitative metric measurements, plaque composition, and PCAT density had no independent or incremental benefit in assessment compared to or in addition to CT-FFR.

## Abbreviations

AS: Aortic valve stenosis
AUC: Area under the curve
CAD: Coronary artery disease
CAD-RADS: Coronary artery diseases reporting and data system
CI: Confidence intervals
CT: Computed tomography
CTA: Cardiac computed tomography angiography
CT-FFR: Cardiac computed derived fractional flow reserve
EAT: Epicardial adipose tissue
FFR: Fractional flow reserve
FOV: Field-of-view
GFR: Glomerular filtration rate
HU: Hounsfield unit
ICA: Invasive coronary angiography
ICM: Iodinated contrast medium
IFR: Instantaneous wave free-period
IQR: Interquartile range
OR: Odds ratio
PCAT: Peri-coronary adipose tissue
RFR: Resting full-cycle ratio
SCCT: Society of Cardiovascular Computed Tomography
TAVR: Transcatheter aortic valve replacement

## Declarations

### Guarantor

The scientific guarantor of this publication is Marcel C. Langenbach.

### Statistics and biometry

One of the authors has significant statistical expertise.

No complex statistical methods were necessary for this paper.

### Study subjects or cohorts overlap

Some study subjects (41 patients, only with CT-FFR) have been previously reported in Wienemann H et al, Feasibility and Comparison of Resting Full-Cycle Ratio and Computed Tomography Fractional Flow Reserve in Patients with Severe Aortic Valve Stenosis (10.3390/jcdd9040116).

### Methodology

• retrospective

• case–control study

• performed at one institution

## References

[1] Sankaramangalam K, Banerjee K, Kandregula K et al (2017) Impact of coronary artery disease on 30-day and 1-year mortality in patients undergoing transcatheter aortic valve replacement: a meta-analysis. J Am Heart Assoc 6. 10.1161/JAHA.117.006092 10.1161/JAHA.117.006092 PMC5721835 29021275

[2] Abdel-Wahab M, Zahn R, Horack M (2012). Transcatheter aortic valve implantation in patients with and without concomitant coronary artery disease: comparison of characteristics and early outcome in the German multicenter TAVI registry. Clin Res Cardiol.

[3] Vahanian A, Beyersdorf F, Praz F (2022). 2021 ESC/EACTS Guidelines for the management of valvular heart disease. Eur Heart J.

[4] Otto CM, Nishimura RA, Bonow RO (2021). 2020 ACC/AHA Guideline for the management of patients with valvular heart disease: a report of the American College of Cardiology/American Heart Association Joint Committee on Clinical Practice Guidelines. Circulation.

[5] Neumann F-J, Sousa-Uva M, Ahlsson A (2019). 2018 ESC/EACTS Guidelines on myocardial revascularization. Eur Heart J.

[6] Svanerud J, Ahn J-M, Jeremias A (2018). Validation of a novel non-hyperaemic index of coronary artery stenosis severity: the Resting Full-cycle Ratio (VALIDATE RFR) study. EuroIntervention.

[7] Andreini D, Pontone G, Mushtaq S (2014). Diagnostic accuracy of multidetector computed tomography coronary angiography in 325 consecutive patients referred for transcatheter aortic valve replacement. Am Heart J.

[8] Meier D, Depierre A, Topolsky A (2021). Computed tomography angiography for the diagnosis of coronary artery disease among patients undergoing transcatheter aortic valve implantation. J Cardiovasc Transl Res.

[9] Chava S, Gentchos G, Abernethy A (2017). Routine CT angiography to detect severe coronary artery disease prior to transcatheter aortic valve replacement. J Thromb Thrombolysis.

[10] Koo B-K, Erglis A, Doh J-H (2011). Diagnosis of ischemia-causing coronary stenoses by noninvasive fractional flow reserve computed from coronary computed tomographic angiograms. Results from the prospective multicenter DISCOVER-FLOW (Diagnosis of Ischemia-Causing Stenoses Obtained Via Noninvasive Fractional Flow Reserve) study. J Am Coll Cardiol.

[11] Wienemann H, Langenbach MC, Mauri V et al (2022) Feasibility and comparison of resting full-cycle ratio and computed tomography fractional flow reserve in patients with severe aortic valve stenosis. J Cardiovasc Dev Dis 9. 10.3390/jcdd9040116 10.3390/jcdd9040116 PMC9030550 35448092

[12] Brandt V, Schoepf UJ, Aquino GJ (2022). Impact of machine-learning-based coronary computed tomography angiography-derived fractional flow reserve on decision-making in patients with severe aortic stenosis undergoing transcatheter aortic valve replacement. Eur Radiol.

[13] Gohmann RF, Pawelka K, Seitz P (2022). Combined cCTA and TAVR planning for ruling out significant CAD: added value of ML-based CT-FFR. JACC Cardiovasc Imaging.

[14] Peper J, Becker LM, van den Berg H (2022). Diagnostic performance of CCTA and CT-FFR for the detection of CAD in TAVR work-up. JACC Cardiovasc Interv.

[15] Goeller M, Achenbach S, Cadet S (2018). Pericoronary adipose tissue computed tomography attenuation and high-risk plaque characteristics in acute coronary syndrome compared with stable coronary artery disease. JAMA Cardiol.

[16] Sezer M, Aslanger E, Cakir O (2021). The Interplay between features of plaque vulnerability and hemodynamic relevance of coronary artery stenoses. Cardiology.

[17] Antonopoulos AS, Sanna F, Sabharwal N et al (2017) Detecting human coronary inflammation by imaging perivascular fat. Sci Transl Med 9. 10.1126/scitranslmed.aal2658 10.1126/scitranslmed.aal2658 28701474

[18] Lin A, Nerlekar N, Yuvaraj J (2021). Pericoronary adipose tissue computed tomography attenuation distinguishes different stages of coronary artery disease: a cross-sectional study. Eur Heart J Cardiovasc Imaging.

[19] Achenbach S, Delgado V, Hausleiter J (2012). SCCT expert consensus document on computed tomography imaging before transcatheter aortic valve implantation (TAVI)/transcatheter aortic valve replacement (TAVR). J Cardiovasc Comput Tomogr.

[20] Blanke P, Weir-McCall JR, Achenbach S (2019). Computed tomography imaging in the context of transcatheter aortic valve implantation (TAVI) / transcatheter aortic valve replacement (TAVR): an expert consensus document of the Society of Cardiovascular Computed Tomography. J Cardiovasc Comput Tomogr.

[21] Leipsic J, Abbara S, Achenbach S (2014). SCCT guidelines for the interpretation and reporting of coronary CT angiography: a report of the Society of Cardiovascular Computed Tomography Guidelines Committee. J Cardiovasc Comput Tomogr.

[22] Coenen A, Kim Y-H, Kruk M (2018). Diagnostic accuracy of a machine-learning approach to coronary computed tomographic angiography–based fractional flow reserve. Circ Cardiovasc Imaging.

[23] Min JK, Taylor CA, Achenbach S (2015). Noninvasive fractional flow reserve derived from coronary CT angiography. JACC Cardiovasc Imaging.

[24] Michail M, Ihdayhid A-R, Comella A (2021). Feasibility and validity of computed tomography-derived fractional flow reserve in patients with severe aortic stenosis: the CAST-FFR Study. Circ Cardiovasc Interv.

[25] Nørgaard BL, Fairbairn TA, Safian RD (2019). Coronary CT angiography-derived fractional flow reserve testing in patients with stable coronary artery disease: recommendations on interpretation and reporting. Radiol Cardiothorac Imaging.

[26] Park H-B, Lee BK, Shin S (2015). Clinical feasibility of 3D automated coronary atherosclerotic plaque quantification algorithm on coronary computed tomography angiography: comparison with intravascular ultrasound. Eur Radiol.

[27] Bittner DO, Mayrhofer T, Puchner SB (2018). Coronary computed tomography angiography-specific definitions of high-risk plaque features improve detection of acute coronary syndrome. Circ Cardiovasc Imaging.

[28] Cury RC, Leipsic J, Abbara S (2022). CAD-RADS™ 2.0 - 2022 Coronary Artery Disease-Reporting and Data System: an Expert Consensus Document of the Society of Cardiovascular Computed Tomography (SCCT), the American College of Cardiology (ACC), the American College of Radiology (ACR), and the North America Society of Cardiovascular Imaging (NASCI). JACC Cardiovasc Imaging.

[29] Shaw LJ, Blankstein R, Bax JJ (2021). Society of Cardiovascular Computed Tomography / North American Society of Cardiovascular Imaging – Expert Consensus Document on Coronary CT Imaging of Atherosclerotic Plaque. J Cardiovasc Comput Tomogr.

[30] Oikonomou EK, Marwan M, Desai MY (2018). Non-invasive detection of coronary inflammation using computed tomography and prediction of residual cardiovascular risk (the CRISP CT study): a post-hoc analysis of prospective outcome data. Lancet.

[31] Oikonomou EK, Antonopoulos AS, Schottlander D (2021). Standardized measurement of coronary inflammation using cardiovascular computed tomography: integration in clinical care as a prognostic medical device. Cardiovasc Res.

[32] Tonino PAL, Bruyne B, Pijls NHJ (2009). Fractional flow reserve versus angiography for guiding percutaneous coronary intervention. N Engl J Med.

[33] Götberg M, Berntorp K, Rylance R (2022). 5-year outcomes of PCI guided by measurement of instantaneous wave-free ratio versus fractional flow reserve. J Am Coll Cardiol.

[34] Lee JM, Choi KH, Park J (2019). Physiological and clinical assessment of resting physiological indexes. Circulation.

[35] Nørgaard BL, Leipsic J, Gaur S (2014). Diagnostic performance of noninvasive fractional flow reserve derived from coronary computed tomography angiography in suspected coronary artery disease: the NXT trial (Analysis of Coronary Blood Flow Using CT Angiography: Next Steps). J Am Coll Cardiol.

[36] Baumann S, Hirt M, Schoepf UJ (2020). Correlation of machine learning computed tomography-based fractional flow reserve with instantaneous wave free ratio to detect hemodynamically significant coronary stenosis. Clin Res Cardiol.

[37] Aquino GJ, Abadia AF, Schoepf UJ (2022). Coronary CT fractional flow reserve before transcatheter aortic valve replacement: clinical outcomes. Radiology.

[38] Williams MC, Kwiecinski J, Doris M (2020). Low-attenuation noncalcified plaque on coronary computed tomography angiography predicts myocardial infarction: results from the Multicenter SCOT-HEART Trial (Scottish Computed Tomography of the HEART). Circulation.

[39] Brandt V, Decker J, Schoepf UJ (2022). Additive value of epicardial adipose tissue quantification to coronary CT angiography-derived plaque characterization and CT fractional flow reserve for the prediction of lesion-specific ischemia. Eur Radiol.

[40] Opolski MP, Kim W-K, Liebetrau C (2015). Diagnostic accuracy of computed tomography angiography for the detection of coronary artery disease in patients referred for transcatheter aortic valve implantation. Clin Res Cardiol.

[41] Naya M, Murthy VL, Blankstein R (2011). Quantitative relationship between the extent and morphology of coronary atherosclerotic plaque and downstream myocardial perfusion. J Am Coll Cardiol.

[42] Hell MM, Dey D, Marwan M (2015). Non-invasive prediction of hemodynamically significant coronary artery stenoses by contrast density difference in coronary CT angiography. Eur J Radiol.

[43] Ferencik M, Mayrhofer T, Bittner DO (2018). Use of high-risk coronary atherosclerotic plaque detection for risk stratification of patients with stable chest pain: a secondary analysis of the PROMISE randomized clinical trial. JAMA Cardiol.

[44] Puchner SB, Liu T, Mayrhofer T (2014). High-risk plaque detected on coronary computed tomography angiography predicts acute coronary syndrome independent of significant stenosis in patients with acute chest pain – results from ROMICAT II Trial. J Am Coll Cardiol.

[45] Wen D, Li J, Ren J (2021). Pericoronary adipose tissue CT attenuation and volume: diagnostic performance for hemodynamically significant stenosis in patients with suspected coronary artery disease. Eur J Radiol.

[46] Zhou K, Shang J, Guo Y (2023). Incremental diagnostic value of radiomics signature of pericoronary adipose tissue for detecting functional myocardial ischemia: a multicenter study. Eur Radiol.

[47] Muthalaly RG, Nerlekar N, Wong DTL (2017). Epicardial adipose tissue and myocardial ischemia assessed by computed tomography perfusion imaging and invasive fractional flow reserve. J Cardiovasc Comput Tomogr.

[48] Tanami Y, Jinzaki M, Kishi S (2015). Lack of association between epicardial fat volume and extent of coronary artery calcification, severity of coronary artery disease, or presence of myocardial perfusion abnormalities in a diverse, symptomatic patient population: results from the CORE320 multicenter study. Circ Cardiovasc Imaging.

[49] Nous FMA, Geisler T, Kruk MBP (2022). Dynamic myocardial perfusion CT for the detection of hemodynamically significant coronary artery disease. JACC Cardiovasc Imaging.

[50] Lu M, Wang S, Sirajuddin A (2018). Dynamic stress computed tomography myocardial perfusion for detecting myocardial ischemia: a systematic review and meta-analysis. Int J Cardiol.

[51] Douglas PS, Pontone G, Hlatky MA (2015). Clinical outcomes of fractional flow reserve by computed tomographic angiography-guided diagnostic strategies vs. usual care in patients with suspected coronary artery disease: the prospective longitudinal trial of FFR(CT): outcome and resource impacts study. Eur Heart J.

